# Compensation for Services

**Published:** 1898-03

**Authors:** W. E. Adams

**Affiliations:** Greensboro, Ga.


### CORRESPONDENCE.

### COMPENSATION FOR SERVICES.

GREENSBORO, GA., February 3, 1898.

*Editors Atlanta Medical and Surgical Journal:*

One of the vital questions which come up for consideration by the doctor in the rounds of his every-day work is how he shall be compensated for his work. It has always been my custom to try and educate my patrons in the duty of paying for service rendered; but in spite of all that can be done, there is a large class of people who never pay for medical service. They change yearly from one doctor to another and in this way avoid paying any one.

The business of this country is larely run on credit, with a mortgage on land, stock, crops, household goods, any and everything that can possibly be mortgaged; so when the doctor is called in to attend them in sickness, he finds his patient has a mortgage covering everything from which he could possibly hope to derive any money. Of course, in a large number of cases the doctor has no time to write and have executed, properly, a mortgage or bill of sale. Now, when the end of the year comes, the doctor is told that the merchant and banker holds a claim on all of their earthly possessions, and he will have to patiently bide his time, and in the wind up he will pay his "doctor's bill." In other words he will pay him out of the "tail end of his crop." So often, oh! much too often the crop has no "tail end," and the hard-worked doctor, who has tenderly, skillfully and attentively looked after him during his sickness, has to go unpaid, and his dear patient and deserving little wife without the comforts and sometimes the necessities of life.

Let me illustrate: John Brown works on the farm of Mr. C, who is both merchant and farmer. He has rented land to John for so much cotton, and agrees to furnish him with the things needful to live while he is making his crop, for which he secures himself by mortgage on all of John's effects and his growing crop. In May John is taken sick with a fever, the doctor, knowing John to be an honest, but poor man, attends hims, furnishes medicine, attendance and all the professional service to restore him back to health. When the fall-time comes and the doctor presents his bill, he is told that it took all of his crop to pay Mr. C's mortgage, and there is nothing out of which to pay the doctor. This is no imaginary picture, but one of too frequent occurrence, as any country doctor well knows.

Now what is the remedy for all this? The merchant, the banker, the carpenter, the landlord, all classes of men are protected by law except our own profession, and I think it high time that we Georgia doctors go before our legislature and ask that we, too, be protected. Give go us a law creating a primary lien on what a man has for a stated amount—say fifteen dollars for every case of sickness attended. Of course I do not mean that this amount in full shall be charged in every case, but we are to be paid according to our bill this amount ($15.00) before any other claim can be satisfied. By this law, if our bill was five or ten dollars, we would be safe; if it was twenty or twenty-five dollars, we would be safe for fifteen dollars. I hope my meaning is clear, and as this is the year when our legislature is to be elected, let every doctor in this State demand a pledge of candidates to vote for some bill which will relieve the financially oppressed doctor. W. E. ADAMS, M.D.

